# Telemedicine-based antibiotic stewardship program in pediatrics: study protocol of a stepped wedge cluster randomized trial—the TeleKasper study

**DOI:** 10.1186/s13063-024-08493-z

**Published:** 2024-10-14

**Authors:** Sophie Diexer, Angelika Ihling, Delphina Gomes, Stefan Moritz, Arne Simon, Christian Dohna-Schwake, Rafael Mikolajczyk, Johannes Huebner, Ulrich von Both, the TeleKasper Consortium

**Affiliations:** 1 Institute for Medical Epidemiology, Biometrics and Informatics (IMEBI), Medical Faculty, Interdisciplinary Centre for Health Sciences, Martin Luther University Halle-Wittenberg, Halle (Saale), Germany; 2 Section of Clinical Infectious Diseases, University Hospital Halle (Saale), Halle (Saale), Germany; 3 Institute of Social Pediatrics and Adolescent Medicine, Division of Pediatric Epidemiology, Ludwig-Maximilians-University Munich, Munich, Germany; 4 Pediatric Oncology and Hematology, University Hospital Homburg Saar, Homburg, Germany; 5 Department of Paediatrics I, Paediatric Intensive Care, Children’s Hospital Essen, Essen, Germany; 6 West German Centre for Infectious Diseases, University Hospital Essen, University Duisburg-Essen, Essen, Germany; 7 Department of Paediatrics, Dr. von Hauner Children’s Hospital, Ludwig-Maximilians-University Munich, Munich, Germany; 8 German Center for Infection Research (DZIF), Partner Site Munich, Munich, Germany

**Keywords:** Antimicrobial stewardship, Telemedicine, Randomized controlled trial, Pediatrics

## Abstract

**Background:**

Overuse and misuse of antibiotics is one of the driving factors of antimicrobial resistance, a growing global health threat. The use of antibiotics is particularly high in children. Even though the implementation of antibiotic stewardship programs (ASP) in pediatrics has been shown to reduce antibiotic use, this implementation has been limited to large university hospitals in Germany. Telemedicine applications might be an effective approach to implement ASP in non-university settings.

**Methods:**

This protocol details the TeleKasper study (Telemedical Competence Network “Antibiotic Stewardship in Pediatrics”). Tele-Kasper is a stepped-wedge cluster-randomized trial that will be conducted across non-university children’s hospitals in Germany. The intervention consists of a telemedical consultation service in the form of a network in different German areas, using an app as a communication tool. The primary outcome will be a 20% reduction in overall antibiotic consumption measured using defined daily doses per 100 patient days.

**Discussion:**

The TeleKasper study aims to implement and evaluate a prototype for a nationwide antibiotic stewardship program by telemedical means in pediatric departments in non-university hospitals in Germany to promote rational antibiotic use and improve medical care for infections.

**Trial registration:**

German Clinical Trials Register (DRKS) DRKS00028534. Registered on 22nd of April 2022.

**Supplementary Information:**

The online version contains supplementary material available at 10.1186/s13063-024-08493-z.

## Background

According to the World Health Organization (WHO), antimicrobial resistance (AMR) is recognized as one of the leading health problems [1]. More than 700,000 people worldwide currently die as a result of antimicrobial resistance and it is estimated that AMR will be associated with the deaths of 10 million people per year by 2050 [2]. Overuse and misuse of antibiotics are some of the driving factors in AMR [1, 3].

This is also the case in pediatric patients, as infections make up a very high proportion of all treatment indications. One of the main causes of this problem is the untargeted, non-restrictive, and non-guideline-based widespread use of antibiotics in inpatient [4] and outpatient [5] pediatric settings.

In 2007, the Infectious Disease Society of America (IDSA) published recommendations for the implementation of antibiotic stewardship programs (ASPs) in pediatrics [6]. This concept has since been realized in some pediatric hospitals [7, 8]. The correct implementation of ASPs in pediatrics has been shown to reduce antibiotic use without compromising treatment safety [9]. However, to date, ASP in the pediatric field have been almost exclusively introduced in large university hospitals. This is mainly because non-university pediatric hospitals are limited in terms of the number of beds and number of staff. Furthermore, ASP for adults cannot be easily adopted to children due to the specific characteristics of pediatric patients. In addition, regional hospitals often lack specifically trained infectious disease personnel to ensure high-quality and efficient care for patients, especially those with complicated infectious diseases [10, 11].

Studies of telemedicine applications of ASP have shown that infectious disease consultations are technically feasible and have good adherence to recommendations when users are actively involved in the development [12]. A recent meta-analysis showed that telemedicine-based ASP increased adherence to guidelines and reduced prescription rates, without affecting mortality rates of patients [13]. Also, in a pediatric setting a telemedicine-based ASP was associated with a reduction of antimicrobial use [14].

The overall goal of this study (Telemedical Competence Network “Antibiotic Stewardship in Pediatrics”—TeleKasper) is to counteract the increasing development of antibiotic resistance and to reduce the risk of possible long-term health consequences of uncritical antibiotic use. This is to be achieved through a comprehensive improvement of infectious patient care and indication-based prescription of antibiotics in the pediatric sector. The primary outcome is the reduction of antibiotic consumption in pediatric inpatient care.

## Methods

This protocol was written based on the Standard Protocol Items: Recommendations for Interventional Trials (SPIRIT) checklist [15]. The completed SPIRIT Checklist is included in the Additional file 1.

### Study design

TeleKasper is a stepped-wedge cluster randomized controlled trial that will implement an antibiotic stewardship program in non-university children’s hospitals in Germany. The overall aim is to provide an antimicrobial stewardship program and consultation service to a selection of non-university pediatric hospitals across Germany, thus establishing the seed for a nationwide ASP network. This aims to improve the infectious diseases care of children across the network and support antibiotic stewardship concepts in smaller, non-university children’s hospitals.

The TeleKasper network will consist of several levels including hospital level, hub level, and network leader level. The hospital level will consist of different non-university hospitals. These individual non-university hospitals will be grouped together into regional hubs. Individual non-university hospitals within each regional hub will be coordinated by a hospital with expertise in pediatric antibiotic stewardship and infectious diseases. All regional hubs are in turn grouped together into a network. The network leader is responsible for the overall coordination of the hubs. In this study, four different demographic regions will be investigated, including areas around Munich, Essen, and structurally less resourceful regions around Homburg/Saar and Halle (Saale). Munich is a prosperous megacity surrounded by an extensive rural area. Essen is a metropolitan area with a concentration of cities with high population density. Homburg is a small University town and Halle (Saale) represents a midsize city both in rather structurally disadvantaged regions.

**Fig. 1:**
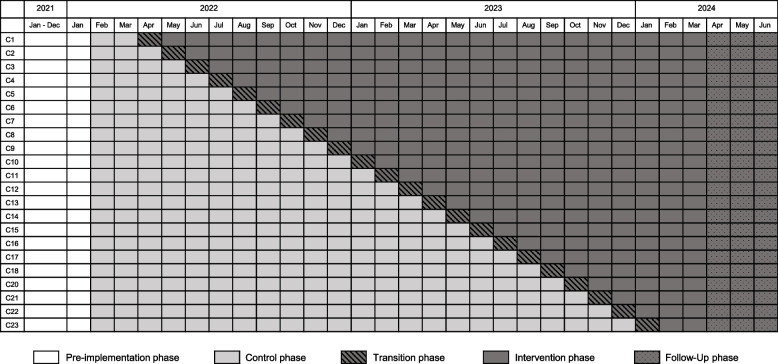
Stepped wedge study design

Non-university hospitals within each hub will be randomized at the beginning of the study for inclusion in the intervention phase. The stepped wedge phase starts with a passive phase of 2 months in which no intervention takes place and the care is as usual. Subsequently, the intervention will start in the first hospital. The plan is to introduce the intervention following a standardized onboarding procedure in the first 2 weeks of the month. However, the entire first month of the intervention will be considered as a transition phase and will not be included in the analysis. Each month, a new hospital will begin the intervention. Every hospital that has undergone the on-boarding process will have a full intervention phase of a minimum of 2 months and will be followed up by an additional follow-up phase of 3 months. During this follow-up phase, the intervention (including recruitment of patients for telemedical consultation via app) will resume and data will be collected, but no new hospitals will be included in the study. The overall intervention phase of the study ends with “the last patient” (June 30th, 2024). Additionally, data will be collected in the pre-implementation phase. This data will be used in the analysis of seasonality effects on antibiotic consumption (Fig. 1).

### Inclusion/exclusion criteria for participating hospitals

All non-university hospitals in the study region (located in the vicinity of one of the four competence hubs including Munich, Essen, Homburg, Halle (Saale)) were eligible. All non-university hospitals eligible for participation had been contacted during the process of TeleKasper grant preparation and provided a letter of intent. In total, 33 non-university hospitals will sequentially be included in the study by their respective competence hubs (10% of all German non-university hospitals). All study sites are listed on the official TeleKasper website [16]. There were no exclusion criteria.

### Intervention

The intervention will consist of a telemedical consultation service within the nationwide network. We will develop a new web-/smartphone-App which will serve as the main tool of this telemedical study. The app will provide a textbook-style reference guide for physicians. This reference guide will include extensive information on infectious diseases and antimicrobial substances including recommended dosages, adverse reactions, and therapeutic drug monitoring (TDM) aspects, among others. In addition, the app includes an innovative communication tool that enables direct interaction between participating hospitals and competence hubs. Via this novel inter-institutional data- and IT-secure network, participating centers are given the ability to make specific inquiries about patients within the network—a telemedical ID consulting service. Depending on the complexity and urgency of these inquiries, app requests can be categorized into the following options: (a) Tele-Info which is a short and general query and is unrelated to any patient directly or indirectly; (b) Tele-Konsil which is a specific patient-related query and is basically a telemedicine ID consultation; (c) a telemedicine case conference in order resolve a complicated query that involves more experts in the discussion; and (d) request for an on-site consultation. Written informed consent from the patient’s parents/legal guardian is required prior to any patient-related query (Tele-Konsil or telemedicine case conference). The informed consent will be obtained from the physicians of the participating non-university hospitals caring for the individual patient. Documents will be placed in a dedicated study folder on-site and stored for a period of 15 years. No patient-identifying information will be stored in the app.

Furthermore, the app serves as an educational tool where presentations on various important antibiotic stewardship topics are presented as live Zoom seminars to all participating centers on a regular basis that can be viewed on demand.

The effect of the intervention will be compared to the present general standard of care and the goal is to demonstrate a superiority compared to the standard of care.

### Randomization

Computer-generated randomization will be used to determine when each hospital will start the intervention. The randomization will be stratified according to the hubs. This has an organizational advantage, as only one hospital per hub is assigned to the intervention group per month. The trial statistician will carry out the randomization after the selection of the study sites. After randomization, the trial statistician will notify the leaders of the respective hubs, who will subsequently inform the hospitals eligible for intervention. If any hospital drops out during the intervention phase, the next hospital will take over.

### Blinding

The hospitals are randomly allocated to pre-determined dates to switch from usual care (control group) to the intervention (intervention group). Due to the open-label design, blinding is not feasible. In this context, data analysts are not blinded either.

### Outcome measures and their assessment

#### Primary outcome

The primary outcome of the study is the antibiotic consumption. This will be measured using defined daily dose (DDD) per 100 patient days. The data will be provided by the hospital pharmacy using anonymized routine electronic health records. In addition, an analysis is also possible for individual departments of the pediatric hospital. All systemic antibiotics will be included according to the second level of the WHO Anatomical Therapeutic Chemical (ATC) classification system for antimicrobials (J01) [17], as well as the substances rifampicin (J04AB02), oral vancomycin (A07AA09), fidaxomicin (A07AA12), and metronidazole (P01AB01). As part of the data management process, an attempt will be made to identify extreme values and, if necessary, corrections will be made in consultation with the corresponding hospital. As the data are part of routine health data, information should not be missing. Nonetheless, missing data will not be imputed or replaced.

#### Secondary outcomes

Secondary outcomes include consumption of specific antibiotic substance groups using DDD per 100 patient days, mean length of stay on pediatric wards, and overall mortality. Furthermore, a secondary objective of the study is also to evaluate the quality of medical care and the accuracy of prescribing. This will be done through 3-monthly point prevalence studies (PPS). All participating hospitals will be eligible to participate in the general pediatric wards PPS. Some centers will also be eligible to take part in a neonatal PPS, which will be conducted every 6 months. Each participating hospital staff must fill in a survey about their current patients, which antibiotics are prescribed, and the (presumed) working diagnosis. No patient-identifying information will be collected by this survey, and the participants will not be allocated an individual trial identification number. The team of the corresponding hub will evaluate this survey based on the following criteria: appropriate use of antibiotic, appropriate duration and dosage of the antibiotic, and if the correct microbiologic diagnosis was performed. Written informed consent for this questionnaire will be obtained from the patient’s parents/legal guardian by the physicians of the participating hospitals. The survey will be distributed via LimeSurvey [18] and the data is stored securely in the network of Martin Luther University Halle-Wittenberg. Finally, the pathogen and resistance data of each hospital will be monitored. This information will be provided by the hospitals or corresponding laboratories.

#### Process evaluation

Technical feasibility and acceptance of the TeleKasper app within the network will be thoroughly evaluated. Parameters of the TeleKasper app usage will include the number of times the app is used by users, frequency of clicks indicating the use of specific app components or sections, frequency of query generation, time taken to answer different types of queries, and rate of reads of specific articles in educational offers. We will also assess the average number of clicks per user. Users will be categorized according to the frequency of use and underlying factors of use will be evaluated. Feedback questions will be asked upon successful completion of Tele-Konsil and Tele-Info to assess query complexity and satisfaction with recommendations related to infectious disease care. The analysis will be performed for the entire network. Subgroup analysis will be performed to evaluate differences between hubs, hospitals, and months during the intervention phase, when appropriate. Users will also be provided a LimeSurvey questionnaire to evaluate the reasons of use and/or no-use of the TeleKasper app.

As part of the process evaluation, an economic evaluation will also be performed comparing cost and cost savings at the hospital level with the costs for providing the novel telemedical ID consult service.

### Sample size and power calculation

For the sample size calculation, we assumed a lead-in phase of 2 months, a stepped wedge phase of 24 months, and a follow-up phase of 3 months. If 35 hospitals are included, assuming an intervention effect of a 20% reduction in antibiotic consumption, and assuming that 60% of the variance in the hospitals’ monthly consumption data is explained by hospital effects, this would result in a power of 83%. Due to a lack of information, possible effects of seasonality are not considered in the power calculation. However, the stepped wedge design allows this to be taken into account in the statistical analysis. With an effect of 20%, a study with 36 hospitals has a power of 84%, and with 32 hospitals 78%. With the 33 participating hospitals the power would be almost 80%.

### Statistical analysis

#### Primary outcome analyses

The primary outcome DDDs per 100 patient days will be measured monthly and analyzed using a Poisson mixed-effects model with an intention-to-treat analysis. The intervention, seasonal effects, and calendar time will be included as fixed effects, and the hospitals will be included as a random effect. Data from the transition phase will be excluded from the analysis. Results will be reported as rates per 100 patient days with 95% confidence intervals.

#### Secondary outcomes analyses

The secondary outcome using DDD per 100 patient days will be analyzed with an analogous approach as the primary outcome. Full information on subgroup analyses of the antibiotic consumption data is provided in the Additional file 2. The other secondary outcomes, including process evaluation parameters, will be analyzed using descriptive statistics. If applicable, the primary method for binary outcomes will be logistic regression and for continuous outcomes linear regression. Data will be analyzed using R software, version 4.2 or higher [19].

### Ethical approval

This study is approved by the ethical committee of the Ludwig Maximilians University under project number 21–0500. The study is registered at the German Clinical Trials Register (DRKS) portal under trial number DRKS00028534.

### Protocol modifications

Any modifications to the protocol will require a formal amendment to the protocol. All protocol modifications will be reported to the sponsor. Such an amendment must be approved by the relevant ethics committee prior to implementation and will then be communicated to the participating non-university hospitals.

## Discussion

Antibiotic consumption in pediatric hospitals is high. ASPs have been shown to reduce antibiotic consumption. This study is a novel approach to implementing an ASP in non-university children’s hospitals by using telemedical means and an innovative app. It is the first nationwide study including 33 non-university hospitals in four different demographic regions in Germany.

This study faced substantial unpredictable challenges and respective evaluation will have to try to account for the effects due to these influencing factors. The COVID-19 pandemic and the associated non-pharmaceutical measures, i.e., lockdown periods, had a notable impact on the incidence of infections, particularly respiratory infections. Once lockdowns had been lifted, both adult and pediatric populations experienced a massive rebound in infections, as described in several publications, including in those from our group [20, 21]. The year 2022 witnessed the most severe RSV season ever to be recorded in Germany as well as in other countries. The severity of the disease led to a substantial increase in antibiotic prescriptions, particularly in very young children [20, 21]. This situation was aggravated by substantial shortages in key antimicrobials both in 2022 and 2023. Subsequently, these shortages affected many first-line antibiotics, such as oral formulation of amoxicillin, jeopardizing any efforts to follow the project’s proposed first-line treatment regimens for a number of pediatric infectious diseases such as otitis media, community-acquired pneumonia or streptococcal pharyngitis.

## Trial status

The randomization of sites was performed in February 2022. The study is currently ongoing, the intervention phase is set to end in March 2024. The last patient for telemedical consultation (Tele-Konsil) will be recruited by June 30th, 2024 (“last patient in”). The team responsible for the study consists of physicians who work as infectious disease experts and were deeply involved in addressing patients’ health needs during the COVID-19 pandemic. Although the plan was to submit the protocol manuscript earlier, competing priorities led to inevitable delays in submission. Protocol Version: 2.0

## Supplementary Information

Supplementary Material 1.

Supplementary Material 2.

## Data Availability

The consortium will have access to the final data. Results and findings of the study will be released through publications in the scientific literature and conference presentations.

## Abbreviations

AMR: Antimicrobial resistance
ASP: Antibiotic stewardship program
ATC: Anatomical Therapeutic Chemical
DDD: Defined daily doses
IDSA: Infectious Disease Society of America
PPS: Point prevalence studies
TDM: Therapeutic drug monitoring
WHO: World Health Organization
