# Supplementary material for: Telemedicine-based antibiotic stewardship program in pediatrics: study protocol of a stepped wedge cluster randomized trial—the TeleKasper study
Source: Trials. 2024 Oct 14;25:678. doi: 10.1186/s13063-024-08493-z (PMC11476715; doi:10.1186/s13063-024-08493-z)
Supplement: Supplementary file 2 — Supplementary Material 2. [file 13063_2024_8493_MOESM2_ESM.docx]

Statistical Analysis Plan

**TELE**medizinisches **K**ompetenznetzwerk „**A**ntibiotic **S**tewardship in **PE**diat**R**ics“

**TELE-KASPER**

Antibiotic consumption data

Content

[**Aim of the analysis plan** 3](#_Toc178197676)

[**Details of the statistical analysis** 3](#_Toc178197677)

[**Outcome** 3](#_Toc178197678)

[**Software** 3](#_Toc178197679)

[**Data Handling** 3](#_Toc178197680)

[**Statistic** 4](#_Toc178197681)

[**Primary outcome analyses** 4](#_Toc178197682)

[**Secondary outcomes analyses** 4](#_Toc178197683)

# **Aim of the analysis plan**

Some assumptions, methods and procedures for the statistical analysis are already described in the study protocol. This analysis plan specifies the statistical and biometric approaches/procedures to be used in detail.

# **Details of the statistical analysis**

**Data basis for the analysis**

The statistical analysis is performed when the stepped wedge phase is completed, i.e. when the last clinic has been included in the study and has been in the intervention phase for two full months. Antibiotic consumption data will be used for the analysis.

## **Outcome**

The following endpoints are considered for the antibiotic consumption data in the statistical analysis:

Primary outcome:

- Defined Daily Dose (DDD) per 100 inpatient days of all antibiotics in the clinic

Secondary outcomes:

- Amount of active ingredient in g per 100 patient days per month
- Recommended Daily Dose (RDD) per 100 patient days per month
- DDD per 100 patient days per month by ward
- DDD per 100 patient days per month Reserve antibiotics
- DDD per 100 patient days per month of various antibiotic groups
  - Broad-spectrum antibiotics
  - 10 most common substances
- Antibiotic consumption in specific substance groups (broad-spectrum antibiotics (carbapenems, linezolides, glycopeptides, 3rd generation cephalosporins) vs. others)
- Ratio of penicillin to cephalosporin consumption

## **Software**

All data will be analyzed using the software R (R Core Team, Vienna, Austria).

# **Data Handling**

Missing data

The following generally applies: Missing information is not replaced, the corresponding value is set to "missing".

Outliers

As part of data management, attempts are made to identify extreme values and corrections may have to be made. Laboratory values are not analyzed in this study, so that corresponding measurement errors do not have to be taken into account.

# **Statistic**

The statistical methods are used to present the recruitment, data quality, homogeneity of the treatment groups and the effectiveness of the treatment. Most evaluations are carried out after the end of the stepped-wedge phase on the basis of the "full analysis set" according to "intention to treat" principles.

Confidence intervals will be reported.

## **Primary outcome analyses**

The statistical analysis is performed using a Poisson mixed effects model (alternative: population average/marginal model with generalized estimating equations (GEE) and "sandwich" standard error (cluster-robust standard error)). In the model, the different clinics are included as random effects. The intervention is considered as a fixed effect. In addition, seasonal effects and the different hospital sizes are taken into account. The seasonal effects are represented by 2 categories (infection season from October to March and non-infection season from April to September). Hospital size is taken into account by means of the number of pediatric beds per hospital.

The primary analysis refers to the stepped wedge design phase.

## **Secondary outcomes analyses**

Further analyses of the primary endpoint also take into account the months before and after the intervention. In addition, it is examined whether there is an effect of the intervention over time, whereby an interaction between the treatment and time is included in the model.

Furthermore, an analysis is carried out that includes the quantitative use of the intervention. Here, the average number of clicks per clinic and the number of consultations per clinic are used.

The analysis of the secondary endpoints of drug quantity in g per 100 patient days, RDD per 100 patient days and the different ways of looking at DDD per 100 patient days is carried out in the same way as the analysis of the primary endpoint. In the ward-based analysis, a distinction is made between three different types of wards (normal ward, neonatology, intensive care unit). The classification of broad-spectrum antibiotics is based on the classification of the surveillance programs.

The ratio of penicillin to cephalosporin consumption is presented and compared before and after the intervention.
